# *In vivo* Recording Quality of Mechanically Decoupled Floating Versus Skull-Fixed Silicon-Based Neural Probes

**DOI:** 10.3389/fnins.2019.00464

**Published:** 2019-05-21

**Authors:** Laetitia Chauvière, Frederick Pothof, Kai S. Gansel, Johanna Klon-Lipok, Arno A. A. Aarts, Tobias Holzhammer, Oliver Paul, Wolf J. Singer, Patrick Ruther

**Affiliations:** ^1^Max Planck Institute for Brain Research, Frankfurt am Main, Germany; ^2^Department of Microsystems Engineering (IMTEK), University of Freiburg, Freiburg im Breisgau, Germany; ^3^ATLAS Neuroengineering bvba, Leuven, Belgium; ^4^BrainLinks-BrainTools Cluster of Excellence, University of Freiburg, Freiburg im Breisgau, Germany; ^5^Ernst Strüngmann Institute for Neuroscience in Cooperation with Max Planck Society, Frankfurt am Main, Germany; ^6^Frankfurt Institute for Advanced Studies, Frankfurt am Main, Germany

**Keywords:** silicon-based neural probes, floating probes, fixed probes, *in vivo* recording, non-human primates, visual cortex

## Abstract

Throughout the past decade, silicon-based neural probes have become a driving force in neural engineering. Such probes comprise sophisticated, integrated CMOS electronics which provide a large number of recording sites along slender probe shanks. Using such neural probes in a chronic setting often requires them to be mechanically anchored with respect to the skull. However, any relative motion between brain and implant causes recording instabilities and tissue responses such as glial scarring, thereby shielding recordable neurons from the recording sites integrated on the probe and thus decreasing the signal quality. In the current work, we present a comparison of results obtained using mechanically fixed and floating silicon neural probes chronically implanted into the cortex of a non-human primate. We demonstrate that the neural signal quality estimated by the quality of the spiking and local field potential (LFP) recordings over time is initially superior for the floating probe compared to the fixed device. Nonetheless, the skull-fixed probe also allowed long-term recording of multi-unit activity (MUA) and low frequency signals over several months, especially once pulsations of the brain were properly controlled.

## Introduction

Chronically stable, extracellular recording of cortical activity is mandatory for brain-computer interfaces used to accurately and reliably control a robotic arm (Velliste et al., 2008; Hochberg et al., 2012). Long-term stable recordings are further required in any neuroscientific study analyzing brain activity at the neuronal level to gain a better understanding of brain dysfunction. Technical tools applied in this context include microwires, i.e., singles wires (Nicolelis et al., 2003) or tetrodes (Gray et al., 1995), flexible, polymer-based probes (Liu et al., 2015; Luan et al., 2017) as well as a variety of silicon-based micro-electrode arrays (Wise et al., 2008; Normann and Fernandez, 2016). While wire electrodes and tetrodes are widely established tools in neuroscientific laboratories which can easily be integrated in multi-channel micro-drives (Lewis et al., 2016), each implanted probe comprises either one or four recording sites only. In contrast, neural probes based on polymeric or silicon substrates comprise a large number of recording sites arranged along slender probe shafts. So far, the number of recording sites of polymer-based probes is limited, however, by the minimally achievable dimensions of metal leads running along the probe shafts. Recent technical developments in case of silicon-based probe arrays (Blanche et al., 2005; Scholvin et al., 2016; Barz et al., 2017) apply sophisticated CMOS-based circuitry integrated directly in the probe shanks (Seidl et al., 2011; Fiáth et al., 2016; Raducanu et al., 2017; De Dorigo et al., 2018; Herbawi et al., 2018). With up to 1600 electrodes along a 10-mm-long probe shaft (Herbawi et al., 2018), a pronounced increase in the number of recording sites is achieved in comparison to any other recording technology.

In view of the long-term recording stability of cortical implants a variety of probe parameters promoting a low tissue response are discussed controversially (Polikov et al., 2005; Karumbaiah et al., 2013; Prodanov and Delbeke, 2016; Salatino et al., 2017). While some studies state that the cross-sectional area, a given surface coating (Rousche et al., 2001), or the tip geometry (Edell et al., 1992) are the main factors influencing the cortical foreign-body response, others negate the importance of these probe characteristics in decreasing the signal quality of neural implants (Szarowski et al., 2003). The mismatch in mechanical properties, i.e., elastic modulus (Nguyen et al., 2014) and material density (Lind et al., 2013), of cortical tissue and neural probe materials was further rated as a possible factor influencing glial scarring and inflammation eventually reducing the long-term recording stability. Responding to the demand for high-density recordings with a large number of simultaneously addressable electrode sites (Dimitriadis et al., 2018) would call for polymer-based probes. Here, the mismatch in mechanical properties of probe material and cortical tissue is minimized (Lacour et al., 2016; Lecomte et al., 2018) resulting in an inherently reduced probe stiffness (Harris et al., 2011). The probe performance is further improved by providing a high level of mechanical decoupling, i.e., by using flexible interconnecting wires (Markwardt et al., 2013).

A histological study by Biran et al. (2007) compared the tissue response caused by silicon-based skull-fixed neural probes and probes floating with the brain. The study clearly indicates a higher immune response from tissue near skull-fixed probes compared to floating implants. It seems further to be widely accepted that mechanical forces acting on neural implants cause a stronger glial response than freely floating devices. Thus, more recent work studying cortical tissue reactions caused by brain implants use by default floating devices (Chestek et al., 2011; Woolley et al., 2013; Ersen et al., 2015). These requirements impose, however, tough constraints on future neural probes that are not easy to fulfill. As an example, advanced CMOS-based probe arrays, as developed by the European project *NeuroSeeker* (Raducanu et al., 2017; Dimitriadis et al., 2018), call for electrically shielded equipment and mechanically stable leads used for the high-frequency data transfer between the probe carrying a large number of recording sites and the external instrumentation. A floating application in a chronic setting would be highly beneficial for *in vivo* recordings from freely behaving animals. However, due to the probe design with a slender probe shaft and a larger probe base, a floating use of this implant is prohibited by the mechanical probe stability. Further, it has been unclear so far whether glial reactions in tissue of non-human primates (NHP) are directly comparable to those in rodents and to which extent glial scarring will prevent the recording of single-unit activity (SUA) from the brain. Recently it has been shown that skull-fixed approaches can be used to record SUA for more than 100 days in mice (Okun et al., 2016) and up to several weeks in a monkey (Lanzilotto et al., 2016).

To find a suitable trade-off between technological requirements of high-frequency circuitry and biological demands regarding glial response, we fabricated a system allowing to directly compare the electrical recording quality of a floating silicon-based neural probe with a skull-fixed device. Both probes consist of the exact same materials and have the same geometry. We implanted and recorded signals over the course of 65 days. Based on the recorded data, we draw a first conclusion regarding the electrical signal quality of the recordings from a NHP (*Macaca fascicularis*).

## Materials and Methods

In order to use both types of probes in close proximity to each other within the same brain area, we fabricated a custom-designed recording chamber to host the floating and skull-fixed neural probes. The silicon-based probes are identical in design; both are 8 mm long and comprise 16 platinum-coated recording sites (diameter 35 μm). They are implanted within a few millimeters of each other.

### Recording Chamber

**FIGURE 1:**
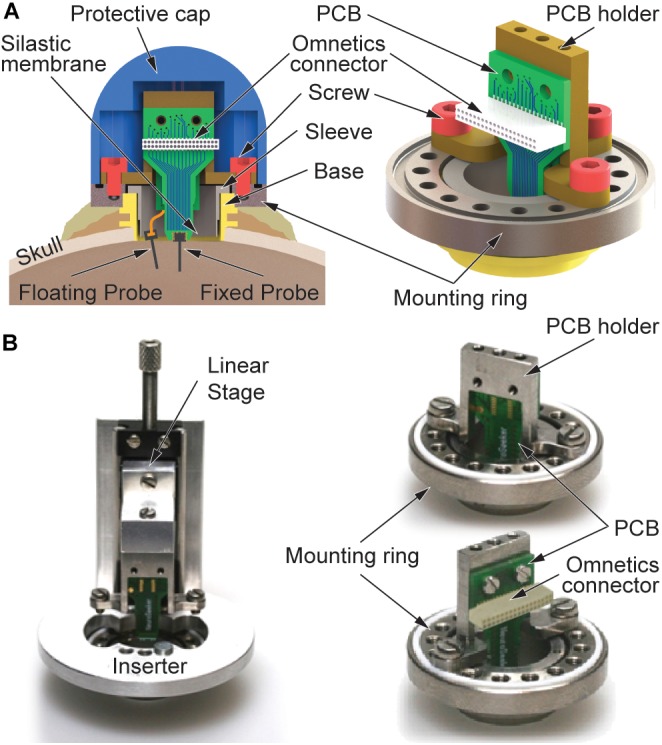
Recording device comprising floating and skull-fixed silicon-based probe arrays. **(A)** Schematic of the recording chamber with mounting frame, PCB holder, and a protective cap. Floating and fixed probes are interfaced with the recording system using an Omnetics connector on the joint PCB. **(B)** Optical images of the mounting frame with (left) inserter used to lower the skull-fixed probe into the brain tissue and (right) fixed PCB holder seen from different perspectives.

Figure 1A shows a schematic of the custom-designed recording chamber. It is based on a commercially available chamber from Gray Matter Research, Bozeman, MT, United States (Gray et al., 2007). The hollow titanium (Ti) cylinder of the chamber with an inner diameter of 12.7 mm is inserted into a circular craniotomy, lowered until it touches the dura mater and fixed to the skull using bone screws and cement. In order to seal the brain surface, a silastic membrane is spun over a sleeve (Figure 1A-left) and lowered to the dura mater using the inner thread of the chamber. In this way, a tight sealing of the brain with respect to the environment is assured. The floating and skull-fixed recording probes will be inserted into the cortical tissue through this silastic membrane and the dura mater. The membrane successfully prevents infections of the brain over a long period of time and blocks body fluids from reaching the electrical connectors inside the recording chamber.

In its upper section, the chamber base (Figure 1A) is equipped with a thread used to fix a mounting ring made of Ti. The mounting ring comprises 16 smaller threaded holes evenly distributed along the perimeter and located on a 20-mm-diameter circle (Figure 1A-right). As indicated in Figure 1A-left, this ring is additionally secured with a second layer of bone cement prohibiting its removal due to excessive torque forces possibly exerted by the freely behaving monkey. As shown in Figure 1B, all custom-designed parts of the novel chamber concept are attached to this mounting ring using screws, i.e., either a linear stage (left) which is temporarily applied during implantation of the skull-fixed neural probe or a holder made of Ti (right) carrying a printed circuit board (PCB) to which the skull-fixed probe is attached. In combination with the 16 threads of the mounting ring, the fact that the probe position on the holder is 1.75 mm off-center provides the possibility to choose different positions of the skull-fixed probe inside the recording chamber. This offers an increased flexibility during probe implantation, enabling to avoid the penetration of larger, visible blood vessels on the brain surface. The entire chamber including its base and mounting ring, together with the PCB holder and respective probe interfaces are mechanically protected by a dome-shaped Ti cap fixed to the mounting ring by four screws.

### Silicon-Based Neural Probes

The silicon (Si) probes used in this study comprise an 8-mm-long probe shaft (width 140 μm, thickness 50 μm) and carry 16 platinum recording sites. These are equidistantly distributed at a pitch of 250 μm over a length of 3.75 mm of the distal shaft section. The probe shaft terminates in a pointy tip which facilitates probe insertion through both the silastic membrane and the dura mater. While the skull-fixed probe is adhesively attached and wire bonded to a PCB mechanically secured on the holder of the recording chamber, we interface the floating probe via a 10-μm-thin polyimide cable with a length of 30 mm (Kisban et al., 2009). The mechanical properties of the cable material, i.e., low elastic modulus, and the geometrical cable dimensions are beneficial in view of minimizing forces caused by brain movements. The flexible cable is connected to the PCB of the skull-fixed probe using a zero-insertion-force (ZIF) connector soldered to the rear of the PCB. Both probes are interfaced to the external instrumentation using a strip connector (NPD series, Omnetics Connector Corp., Minneapolis, MN, United States). The probe fabrication using microsystem technologies has been described in detail elsewhere (Herwik et al., 2011).

### Implantation

For implantation of the recording chamber, a trepanation fitting to the diameter of the chamber base is cut into the skull of the monkey at the desired position. Bone screws are in addition implanted into the skull close to the chamber. They are used to fix the chamber base to the skull using acrylic bone cement. The brain surface with intact dura mater is sealed using the silastic membrane mechanically fixed using the Ti sleeve. The skull-bone cement interface is allowed to mechanically strengthen in a subsequent healing period of 3 weeks before the probes are implanted through the silastic membrane and the dura mater. In this period, the chamber is closed by a flat cap from Gray Matter Research, effectively sealing the chamber.

Using a vacuum inserter, the floating probe is inserted under microscope control into the targeted brain area, as previously described in Bonini et al. (2014). If needed, the inserter provides the possibility to retract and reposition the floating probe at a different position within the chamber. The skull-fixed probe assembled on a PCB is slowly lowered into the cerebral cortex using the inserter temporarily fixed on the mounting ring, as shown in Figure 1B-left. The inserter comprises a manually operated linear stage. The PCB holder together with the probe-carrying PCB are connected to this linear stage using screws. Before lowering the PCB-mounted probe into the cortical tissue, the floating probe is electrically connected to the PCB using the ZIF connector on the PCB rear. As in the case of the floating probe implantation, lowering the PCB-mounted probe using the linear stage causes the silastic membrane and the dura mater to be penetrated. The penetration of both layers is facilitated by the beveled probe tip. Maximal implantation depth is reached once the PCB holder is in contact with the mounting ring. At this point, the PCB holder is fixed to the ring using three screws, followed by the removal of the inserter, as depicted in Figure 1B-right with front and rear views of the holder. The electrophysiological recordings start the next day, once the animal has fully recovered from anesthesia.

The study was conducted with one adult macaque (*Macaca fascicularis*) following the guidelines of the European Community for the care and use of laboratory animals (European Union Directive 86/609/EEC) with approval by the appropriate local committee on animal welfare (Regierungspräsidium Hessen, Darmstadt, Germany). During the recordings of this study, the monkey was exclusively in the awake state while receiving rewards for fixating his eyes on a fixation spot at the center of a monitor screen [cf. methods section of Chauvière and Singer (2019)].

### Electrophysiology and Data Analysis

The electrophysiological recordings of the study were performed using a recording system from Tucker Davis Technologies (RZ2 Z-series processor, Tucker Davis Technologies, Alachua, FL, United States). We sampled broadband activity at 25 kHz which was band-pass filtered between 1 and 300 Hz for local field potentials (LFPs) and between 300 and 3000 Hz for multi-unit activity (MUA). The recorded data were stored offline, and sessions were concatenated before sorting the recorded spiking activity in all the sessions at once using a custom-made spike sorter based on a dynamic template matching algorithm. Statistics have been performed using the ANOVA test, with further *post hoc* testing using Dunnett’s test.

### Spike Sorting Analysis

Offline spike sorting was performed using a dynamic template matching method implemented in a custom software package (“Smart Spike Sorter”). Initially, up to twelve different clusters were automatically defined by an artificial neural network based on the adaptive resonance theory (Carpenter and Grossberg, 1987). Various cluster properties like auto-correlations of spike times and recording stabilities of spike waveforms were monitored and considered in conjunction with the shape of the waveforms to guide decisions about which clusters to merge or delete. Only clusters visibly separated in 3D principal component space were assigned to single units. Accuracy of spike assignment was validated by objective measurements of cluster separation provided by the J3 and Pseudo-F statistics. Based on these criteria, only well-isolated putative single units were considered for further analysis.

### Behavioral Paradigm and Passive Viewing Task

**FIGURE 2:**
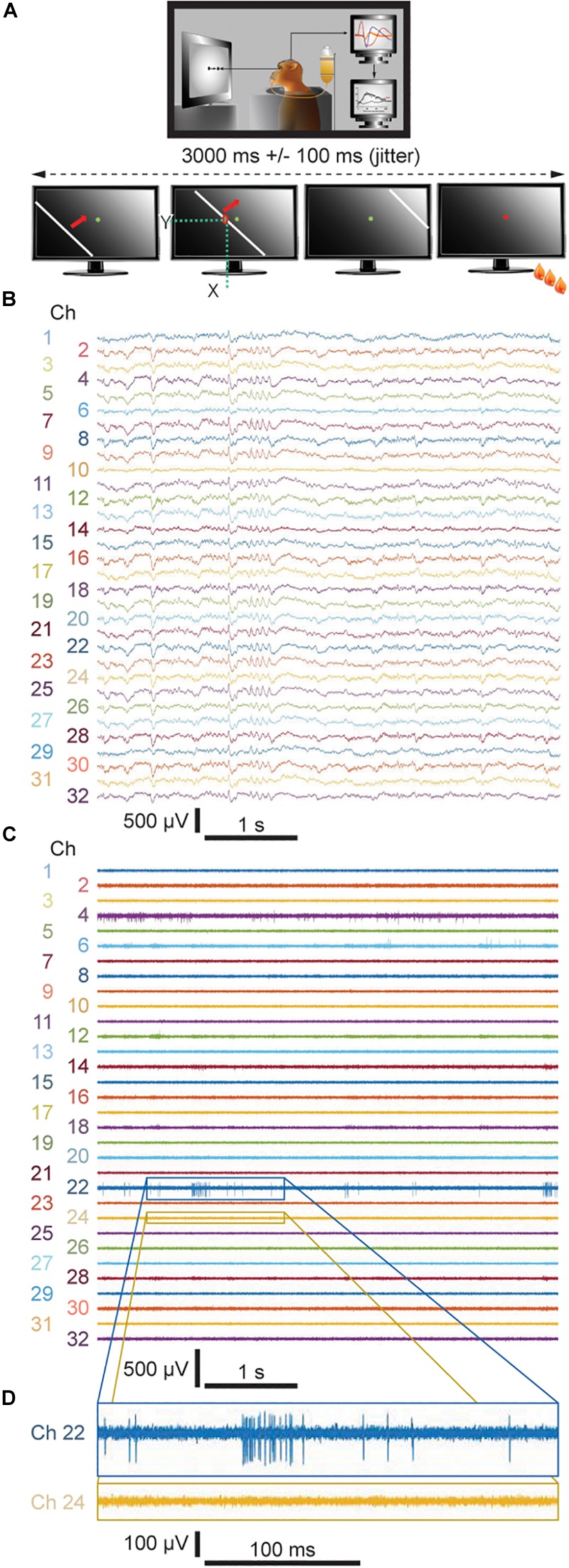
**(A)** Visual task applied during the recording sessions. **(B)** Representative 5-sec-long traces of LFP signals downsampled to 1525.9 Hz from both the fixed and floating probes recorded during Week 8. **(C)** Representative 5-sec-long traces of unit activity from both the fixed and floating probes bandpass filtered between 300 and 3000 Hz and **(D)** 500-ms-long enlarged views of two representative channels. **(B,C)** even numbers: floating probe; odd numbers: skull-fixed probe.

As described in Figure 2A, in this study the monkey had to perform a passive viewing task. The task consists of pressing and releasing a lever in response to the color change of a light dot shown at the center of a monitor screen. In order to initiate a trial, the monkey was trained to press the lever. This is followed by the appearance of the green fixation spot at the center of the monitor screen. The monkey has to keep fixation and the lever pressed until the point changes color (e.g., from green to red). If the monkey has kept fixation and released the lever within a short interval after the color change it gets a reward (a drop of water or juice). An error signal (i.e., a sound) is presented in case the monkey does not press the lever, does not maintain it pressed, does not fixate the color spot or releases the lever too late or too early. In these cases, the monkey does not get a reward. Eye movements were recorded with an infra-red eye tracker (Thomas Recording, Giessen, Germany) to assure that the monkey maintained fixation within a 0.5° window of visual angle. If the monkey broke fixation before the fixation spot changed color the trial was aborted.

During the viewing task, a white bar with 16 different, predefined orientations evenly distributed over 360°, travels across the monitor screen within 3 s with an added jitter of 100 ms (Figure 2A, lower schematics). Spike amplitude above a given threshold, i.e., 3 times the standard deviation from the noise level, was recorded at each channel for each position of the bar. The 16 directions are randomly repeated 10 times per recording session and responses to the same stimulus were averaged. The receptive fields were determined for each channel by adding the number of spikes on the respective channel at the moment when the bar passes by an individual pixel (*x* and *y* positions) on the monitor screen (Figure 2A). For each channel of the probe, the boundaries of the receptive fields correspond to the location of the bar where it starts to induce increased discharges.

## Results

### Comparison of Neuronal Activity Between the Two Types of Silicon-Based Neural Probes

Over the course of 8 weeks, neuronal activity was recorded within the primary visual cortex of one awake behaving monkey. We applied one floating and one skull-fixed silicon-based neural probe implanted within the same recording chamber, while the monkey was performing a simple passive viewing task (Figure 2A; cf. Methods section for a detailed description of the behavioral paradigm). We then compared the neuronal activity from both types of probes and found that LFPs could be recorded from both probe types already 1 day after probe implantation (Figure 2B where exemplary signals are from recordings during Week 8). In the raw signal traces, a difference between floating and fixed probe cannot be discerned (floating probe, even numbers; fixed probe, odd numbers). The LFP signals were stable throughout the entire observation period (cf. Supplementary Figure S1 for similarity measures between LFP signals recorded by means of both types of probes and Supplementary Table S1 for their quantification).

### Spiking Activity

**FIGURE 3:**
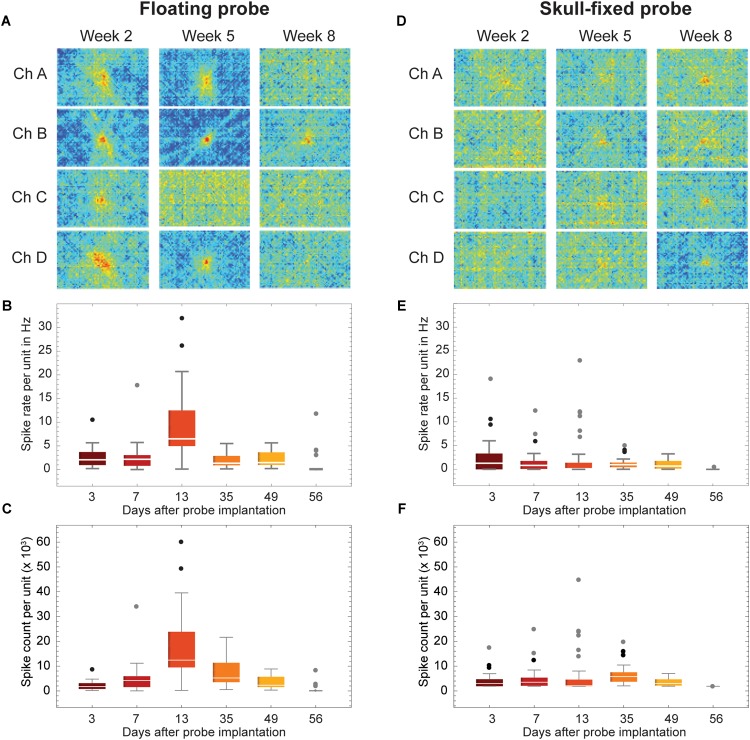
Recordings stability across weeks of recordings. **(A,D)** Examples of visual response stability, i.e., receptive fields, across weeks of recordings (2 weeks, 5 weeks, and 8 weeks post-insertion of the probes). **(B,E)** Spike rate and **(C,F)** spike count of all units for selected recording sessions. Data is grouped for **(A–C)** floating and **(D–F)** skull-fixed neural probes (black circles: outlier, outside 1.5× interquartile range; gray circles: far outlier, outside 3× interquartile range).

Comparing MUA between the floating and skull-fixed probe, we found that 3 days post-implantation, MUA was identifiable from the floating probe (Figure 2C, even numbers), providing reliably mappable receptive fields for Week 2 and Week 5 (Figure 3A).

Retesting the animal at regular time intervals revealed that the floating and the skull-fixed probes both reliably recorded MUA activity from varying cell populations over the first five sessions (Figure 3B,C for the floating probe, Figure 3E,F for the skull-fixed probe). However, some differences were noted: while in the third session, i.e., 13 days after probe implantation, the floating probe yielded higher spike counts, those from the fixed probe remained unchanged. For both probes, however, MUA activity decreased over the last (sixth) session (ca. 8 weeks after implantation). Comparing the spike counts (or rate) obtained from the fixed and the floating probe, respectively, we found that the former represented ∼98.2, 55.1, 24.9, 66.1, 51.6, and 0.1% of the latter during the recording sessions 3, 7, 13, 35, 49, and 56 days after implantation, respectively. The receptive fields were mappable for both probes (Figure 3A,D), but spikes varied in amplitude for the different recording sites, sometimes barely exceeding the noise level at some of the recording sites (Figure 2). Overall, however, the variability between recording channels of the same electrode exceeded the variability among the two probes.

### Unit Activity

**FIGURE 4:**
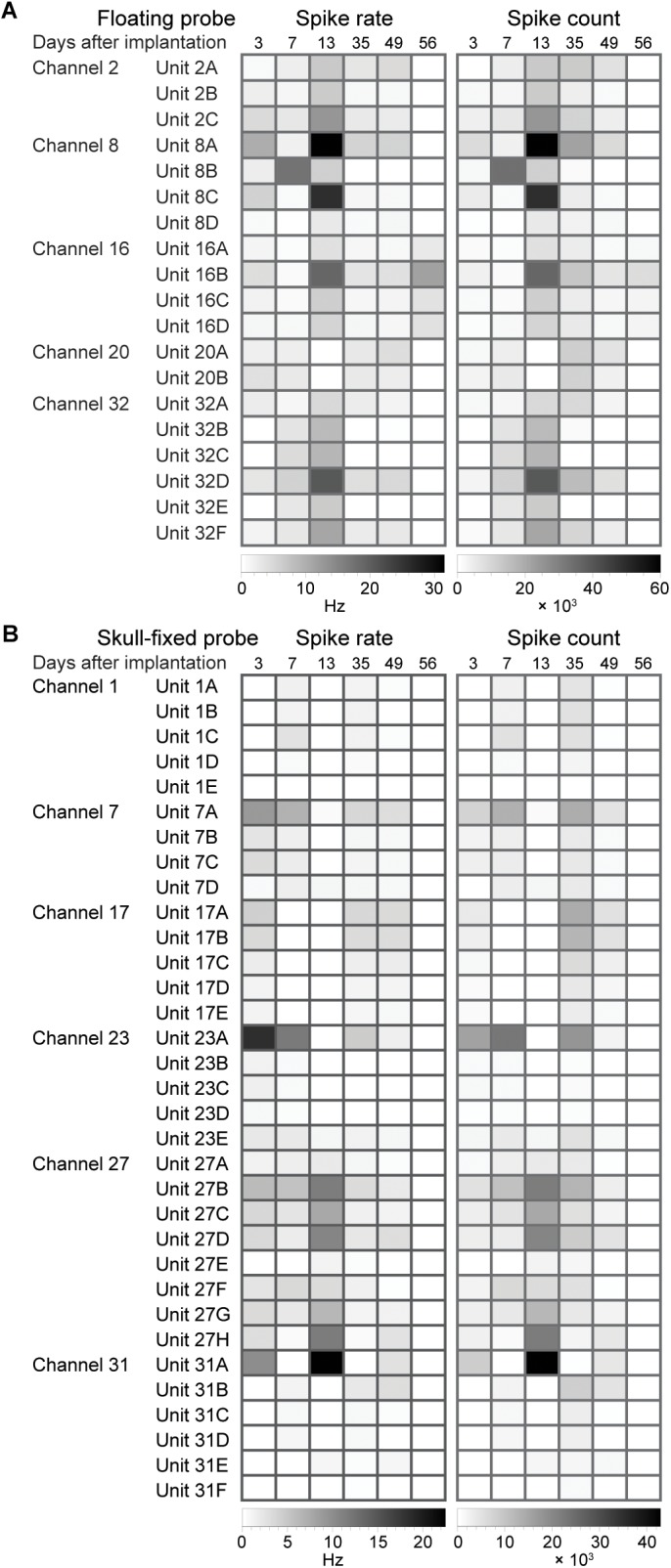
Evolution of spike statistics across units and over weeks of recordings. Spike rate and spike count for **(A)** floating and **(B)** skull-fixed probes for different channels and units sorted on the specific channel.

**FIGURE 5:**
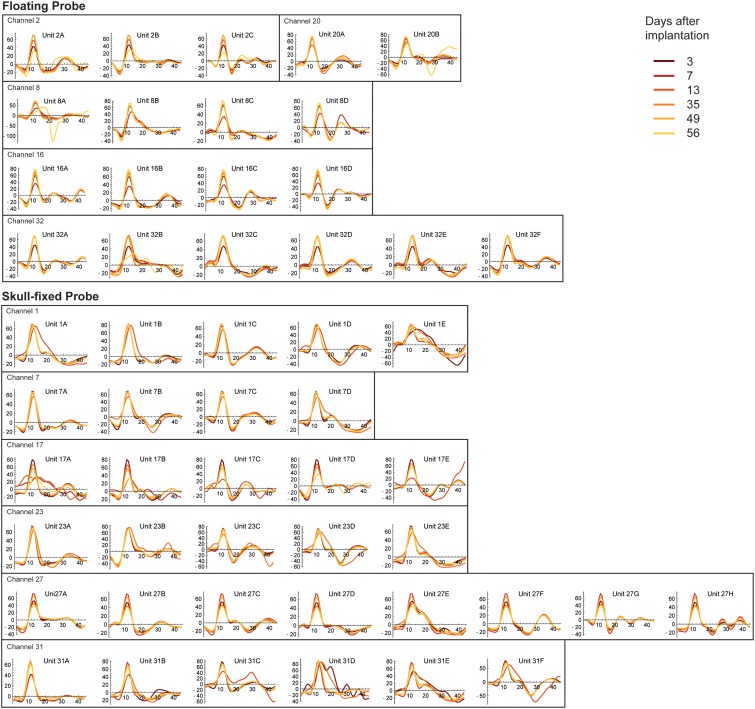
Stability of spike waveforms across units, recording sites, and recording sessions. The *y*-axis displays spike amplitude (in μV), the *x* axis displays time, expressed in samples. As the sampling frequency of our recording system was 24414 Hz, one sample corresponds to 40.96 μs (therefore 44 samples correspond to ∼1.8 ms). Note units displaying stable waveforms and spike widths with stable (Channel 7 of the fixed probe, Channel 20 of the floating probe) or varying spike amplitudes (Channel 2 of the floating probe and Channel 17 of the fixed probe) along different recording sessions.

**FIGURE 6:**
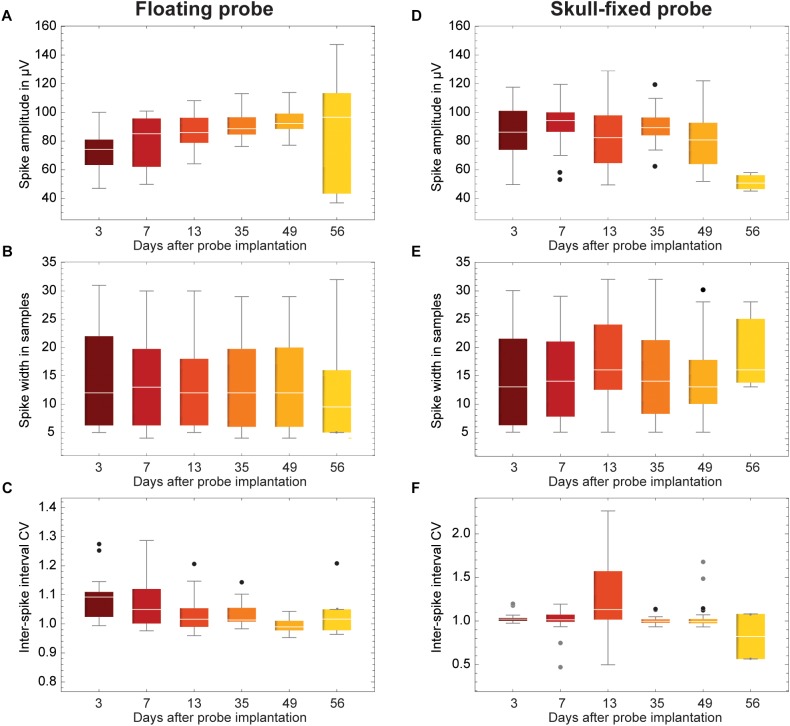
Evolution of spike statistics across units and over weeks of recordings. **(A,D)** Spike amplitude; **(B,E)** Spike width; and **(C,F)** Coefficient of variation of inter-spike interval (ISI CV). Data is grouped for **(A–C)** floating and **(D–F)** skull-fixed neural probes (black circles, outlier, outside 1.5× interquartile range; gray circles, far outlier, outside 3× interquartile range).

For each of the recording channels, spikes were sorted on concatenated recording sessions and split into putative single units according to their waveforms using a dynamic template matching algorithm allowing for slow drifts in spike amplitude of a single unit. We isolated 2 to 8 units per recording site, with an average of 4 units per site for the floating probe and of 5–6 units for the skull-fixed probe (see details in Figure 4). We analyzed in further detail a total of 19 units for the floating probe and 33 units for the fixed probe which displayed overall more units per recording site. For this in-detail analysis, a total of 52 units over a period of 8 weeks following probe insertion were thus considered. We studied the temporal stability of the units across different recording sessions, displayed in Figure 5. For each unit and session we analyzed the spike rate and spike count (Figure 4), the spike amplitude (Figure 6A,D), the spike width (Figure 6B,E), and the inter-spike interval coefficient of variation (ISI CV, Figure 6C,F) for the floating probe (Figure 4A, 6A–C) and the skull-fixed probe (Figure 4B, 6D–F). Table 1 summarizes the statistical results for the comparisons of the sessions (ANOVA).

### Spike Amplitude and Spike Width

Overall, the measured peak-to-peak spike amplitudes varied between 40 and 150 μV. For the fixed probe, the amplitude remained rather stable over the recording sessions, except for the last session when the amplitude clearly dropped (Figure 6D and Table 1). For recordings with the floating probe, a slight but steady increase of the average spike amplitude could be detected over the entire course of the recording sessions (Figure 6A and Table 1). The interquartile range is, however, strongly increased for the sixth recording session. The individual spike widths were quite stable along the whole recording period (53 days) for both the floating and the fixed probe (*p* > 0.05 for both probes; see Table 1 and Figure 6B,E).

### Stability of Spiking Activity Over Recording Sessions

To illustrate the stability of spiking activity over recording sessions for both probe types, examples of units displaying stable waveforms and spike widths with stable (Channel 7 of the fixed probe, Channel 20 of the floating probe) or varying spike amplitudes (Channel 2 of the floating probe, Channel 17 of the fixed probe) along different recording sessions are shown in Figure 5. Altogether, these results showed that the quality of the recorded signals was good and fairly stable over 8 weeks following probe insertion for both types of probes. However, in the last session (8 weeks post-implantation) recording quality had decreased as indicated by the drop in MUA activity.

### Coefficient of Variation and Inter-Spike Interval

Finally, the ISI CV was close to one in most instances (Figure 6C,F), showing a significant excursion only for recordings with the fixed probe during the third session (Figure 6F), indicating less regular firing at a constant overall firing rate.

## Discussion

### Stability of Recordings With Chronic Laminar Probes

In the present study we assessed the stability of long-term recordings from chronically implanted silicon-based laminar probes. Stability criteria were the ability to record spiking activity (MUA), the drift in the shape of action potentials analyzed after spike sorting and application of template matching procedures and the signal-to-noise ratio of LFPs. In agreement with previous publications we found that LFP recordings were stable over the whole analysis period (Andersen et al., 2004; Flint et al., 2013; Hall et al., 2014) but recordings of spiking activity exhibited both short and long-term variability. The probability to pick up well isolatable unit discharges changed from session to session for individual recording channels of both probes and spike shape analysis suggested changes in the relative position of recording sites and contributing neurons. This is expected since the brain can move substantially due to changes of intracranial pressure caused by respiration, coughing, heart beat and changes in posture, and as a consequence of rapid head movements. In addition to these short-term and mostly reversible changes in recording conditions we observed a gradual deterioration of the ability to record MUA responses that became manifest 8 weeks after implantation.

**Table 1:** Summary of ANOVA statistics together with Dunnett’s *post hoc* tests for spike rate per unit, spike amplitude, spike width and ISI CV comparing recording sessions for both types of probes.

		DF^1^	Sum of Sq	Mean Sq	F Ratio	*p* Value
**Spike rate (per unit and per session)**						
**Floating probe**						
	Model	5	977.776	195.555	10.965	1.499 × 10^−8^
	Error	108	1926.16	17.835		
	Total	113	2903.93			
Post tests	Dunnett: session 3 significant					
**Fixed probe**						
	Model	5	146.58	29.316	3.673	3.384 × 10^−3^
	Error	192	1532.48	7.982		
	Total	197	1679.06			
Post tests	Dunnett: session 6 significant					
**Spike amplitude (per session)**						
**Floating probe**						
	Model	5	5707.25	1141.45	4.017	2.321 × 10^−3^
	Error	99	28129.5	284.136		
	Total	104	33836.8			
Post tests	Dunnett: sessions 3, 4, 5 significant					
**Fixed probe**						
	Model	5	6961.07	1392.21	4.978	3.020 × 10^−4^
	Error	150	41955	279.7		
	Total	155	48916.1			
Post tests	Dunnett: session 6 significant					
**Spike width (per session)**						
**Floating probe**						
	Model	5	24.430	4.886	0.068	0.997
	Error	99	7089.53	71.611		
	Total	104	7113.96			
**Post tests**	Dunnett: no session significant					
**Fixed probe**						
	Model	5	242.815	48.563	0.828	0.532
	Error	150	8798.33	58.656		
	Total	155	9041.15			
Post tests	Dunnett: no session significant					
**ISI coefficient of variation**						
**Floating probe**						
	Model	5	0.131	0.026	2.185	0.062
	Error	95	1.139	0.012		
	Total	100	1.270			
Post tests	Dunnett: session 5 significant					
**Fixed probe**						
	Model	5	8.159	1.632	6.755	1.097 × 10^−5^
	Error	144	34.782	0.242		
	Total	149	42.941			
Post tests	Dunnett: session 3 significant					

*^1^DF: degrees of freedom.*

### Comparison of Recording Stability Between Floating and Fixed Silicon-Based Laminar Probes

The comparison between the recording performances of a floating and a skull-fixed silicon-based neural probe, inserted in the same custom-made chamber, demonstrated that both implantation types are suitable to obtain long-term stable recordings of LFP and MUA from the primary visual cortex of awake behaving monkeys. The floating probe yielded slightly more stable recordings with better signal-to-noise ratios right after implantation and during the first weeks. The likely and anticipated reason for the higher recording performance variability of the skull-fixed probe is movements of the brain relative to the fixed probe. Such movements can account for the transient loss of spiking activity from individual recording sites and changing composition of neurons contributing to the recorded MUA. In addition, it is likely that the mechanical irritation caused by the movement induced glial reactions that may have further reduced recording performance (Ward et al., 2009). However, the differences between the floating and the fixed probe diminished with time because the recordings from the fixed electrode became more stable and at the end of the recording period, signal-to-noise ratios had decreased for both probes at some recording sites. This gradual degradation is expected and likely results from glial proliferation and chemical processes deteriorating the interface between the electrodes and the nervous tissue. We cannot differentiate between the two processes but apparently they had affected both probes, similarly. However, we anticipate that the yield of the skull-fixed probe can be increased substantially, in particular over long durations, if mounted on a microdrive permitting further adjustments – an option not available for floating probes (see below).

### Spike Sorting

If spikes have similar waveform but are recorded from different sites with single electrodes, it must be assumed that they come from different neurons. However, spikes with similar waveforms recorded in the same session from the same electrode are likely to originate from the same cell, in particular if there are indications for a refractory time in the interval distribution. The recording horizon of a single electrode typically includes only up to twenty neurons in cortical layer V and fifty neurons in layer IV, the majority of cells tend to be most of the time silent and numerous cells with small spikes escape sorting. Thus, chances that more than one neuron contributes to a collection of identical spikes are reasonably low. Whether spikes with similar waveforms recorded from the same channel but in different sessions are from the same cell is impossible to decide when recordings are discontinuous as in the present experiment. Thus, we ignore for how long individual cells can be followed.

### Firing Rates

The spontaneous firing rates of the sampled neurons were low as is characteristic for cortical neurons. They increase to variable extents with light stimulation. However, the increase depends critically on the type of neurons and the match between stimulus configuration and receptive field structure. In the present experiments no attempts were made to optimize this match. For the determination of basic receptive field properties (position, orientation preference, and direction preference) we used simple grating stimuli and light bars, whose orientation and direction of drift were varied systematically. Thus, it is conceivable that we failed to optimally drive some of the recorded cells. However, we had no indications for pathological discharge patterns such as sustained high frequency discharges or high frequency bursts.

### Potential Explanations for Recording Stability Variations

The gradual increase in recording stability observed for the fixed probe is probably due to several processes that reduced relative movements between the brain and the probe. Insertion of probes is inevitably associated with dimpling of brain tissue due to compression, and the gradual re-expansion of tissue that may last several days leads to relative motion and unstable recordings. The floating probe is less exposed to this effect as it moves with the brain. Another factor for a protracted increase in stability is improved sealing. After craniotomies the exposed dura mater tends to proliferate. This has the dual effect that the leakage of cerebrospinal fluid through the hole caused by the penetrating probe gets sealed and that the stiffness of the dura increases. Both factors reduce movements of the brain and thereby enhance recording stability. Again, the floating probe is less likely to benefit from these proliferative processes which likely account for the time-dependent decrease of the differences in performance between the two probes (Santhanam et al., 2007). As expected from numerous previous studies we observed neither a time-dependent change in the ability to record LFPs, nor did we see differences between the two probes. LFPs reflect the activity of large populations of neurons, and hence are little influenced by small movements nor by changes in the micro-environment of the neural probe (Andersen et al., 2004; Flint et al., 2013; Hall et al., 2014).

The fact that spikes get smaller across sessions probably results from a deterioration of the interface between the electrode and the tissue (glial reactions, oxidation of the electrode, etc.). That spikes become wider could mean that electrodes become less sensitive and pick up only cells with large dipoles and these tend to be large pyramids with broad spikes.

Numerous studies have shown that long-term recordings can be obtained with skull-fixed, chronically implanted microwires, or matrix electrodes in rodents (Karumbaiah et al., 2013; Kozai et al., 2015), NHP (Jackson and Fetz, 2007; Tolias et al., 2007; Hall et al., 2014; McMahon et al., 2014) and human subjects (Serruya et al., 2002). However, in cases where the electrodes could not be moved once implanted, there was always a gradual degradation of the ability to record spiking activity. Most likely this is not due to a general deterioration of the electrodes because small movements of the electrode can often reinstall satisfactory recording conditions (cf. method section of Gray et al. (2007). Thus, the likely reason for degradation are changes in the micro-environment in the immediate vicinity of the electrode tips or the hot spots in case of silicon probes. Such adjustments are routinely performed in rodent experiments with chronically implanted hyperdrives that allow continuous advancement of micro-wire electrodes (tetrodes) and permit good recordings over months. Similarly, successful long-term recordings have been reported in NHPs implanted with the multi-electrode drive from Gray Matter Research, that permits continuous depth adjustments of 32 independently controlled glass-coated platinum-iridium or tungsten microelectrodes. We work with Gray Matter Research drives and can obtain MUA activity from the large majority of electrodes even 4 years after implantation (personal observation). In our experience, small movements of the electrodes (∼50 μm up or down) suffice to recuperate MUA recordings.

### Conclusion

In conclusion, our results suggest a slight advantage in recording stability of floating over fixed probes in early phases after chronic implantation but this advantage levels off after several weeks. Therefore, a number of arguments lead us to suggest to not pursue the floating solution any further, but to rather invest in the perfection of chronic implantation of head fixed high-density laminar probes in NHPs. A major disadvantage of floating probes is that they cannot be adjusted after insertion which limits the possibility to optimize the number of hot spots recording MUA responses. Another problem is their connection with the plug in the chamber. Already with our 16-channel probes, these connections required the development of special highly flexible ribbon cables. When the number of hot spots is scaled up to several hundreds, this solution becomes obsolete because the cables become too rigid. The alternative to realize connections via a PCB is equally incompatible with a floating solution.

### Future Directions of Research

In the light of this evidence the results obtained in the present study with laminar probes are promising and suggest the following strategy for the chronic implantation of high-density laminar probes. We suggest opting for a skull-fixed configuration and the use of a PCB for connections as described in this study. Leaving the dura mater intact and assuring a hermetic seal of the intracranial space with an additional silastic membrane proved sufficient to prevent loss of cerebrospinal fluid and infections. However, this approach required beveling of the tip of the laminar probe in order to permit penetration of the two barriers. To overcome the initial instability of recording performance and to minimize tissue irritation by movement-related friction it is imperative to further reduce brain movement. In a pilot experiment, we have therefore begun to test a viable and simple solution for the reduction of pulsations. We enlarged the base plate of the inserter, brought it in direct contact with the silastic membrane and advanced the electrode through a small hole in the baseplate. This effectively reduced brain pulsation and assured stable recordings right from the beginning. Furthermore, we suggest to miniaturize the inserter so that it can remain in the chamber and permit repeated adjustment of the probe position. In this case the probe can be moved up and down without further surgical intervention. In this way the distance of the hot spots relative to active neurons can be optimized and neuronal responses recovered over very long periods of time. In addition, the probe can be retracted once laminar recordings have been completed, the inserter can be removed together with the electrode and replaced by other devices that permit insertion of electrode arrays covering larger regions. All these manipulations can be executed without breaking the seal between the chamber and intracranial space and without surgery. This should permit long-term recordings with changing electrode configurations and will substantially enhance the wealth and quality of data obtainable from a single animal. As NHP can learn to perform a great variety of tasks and usually cooperate over many years, numerous different paradigms can be investigated in the same animal following a single surgical intervention for the implantation of the recording chamber.

We are currently investigating these options further and hope to be able to report about the results in the near future. If successful, this approach would pave the way for the chronic implantation of various electrode configurations, including high density laminar probes and would substantially scale up the amount of data obtainable per animal, thereby complying with two of the three R’s (reduce, refine, and replace) which are mandatory for the protection of experimental animals.

## Author Contributions

PR and WS developed the concept and design of the study. LC was involved in the feasibility of the study in monkeys, organized the database, analyzed the LFP data, and performed spike-sorting. KG performed the statistical analysis on spike-sorted data. JK-L performed the *in vivo* recordings. FP realized the modified recording chamber and designed the probe interfaces. AA, TH, and OP participated in the technical aspects of the probe design. LC wrote the first draft of the manuscript. LC, FP, PR, and WS wrote sections of the manuscript. All authors contributed to manuscript revision, read and approved the submitted version.

## Conflict of Interest Statement

TH, AA, OP, and PR are co-founders of ATLAS Neuroengineering bvba, Leuven, Belgium. TH and AA are CTO and CEO of ATLAS, respectively. The remaining authors declare that the research was conducted in the absence of any commercial or financial relationships that could be construed as a potential conflict of interest.
