# Supplementary material for: In vivo Recording Quality of Mechanically Decoupled Floating Versus Skull-Fixed Silicon-Based Neural Probes
Source: Front Neurosci. 2019 May 21;13:464. doi: 10.3389/fnins.2019.00464 (PMC6536660; doi:10.3389/fnins.2019.00464)
Supplement: Supplementary file 1 [file Image_1.pdf]

Recording session of Week 8
All trials (n = 53 trials)

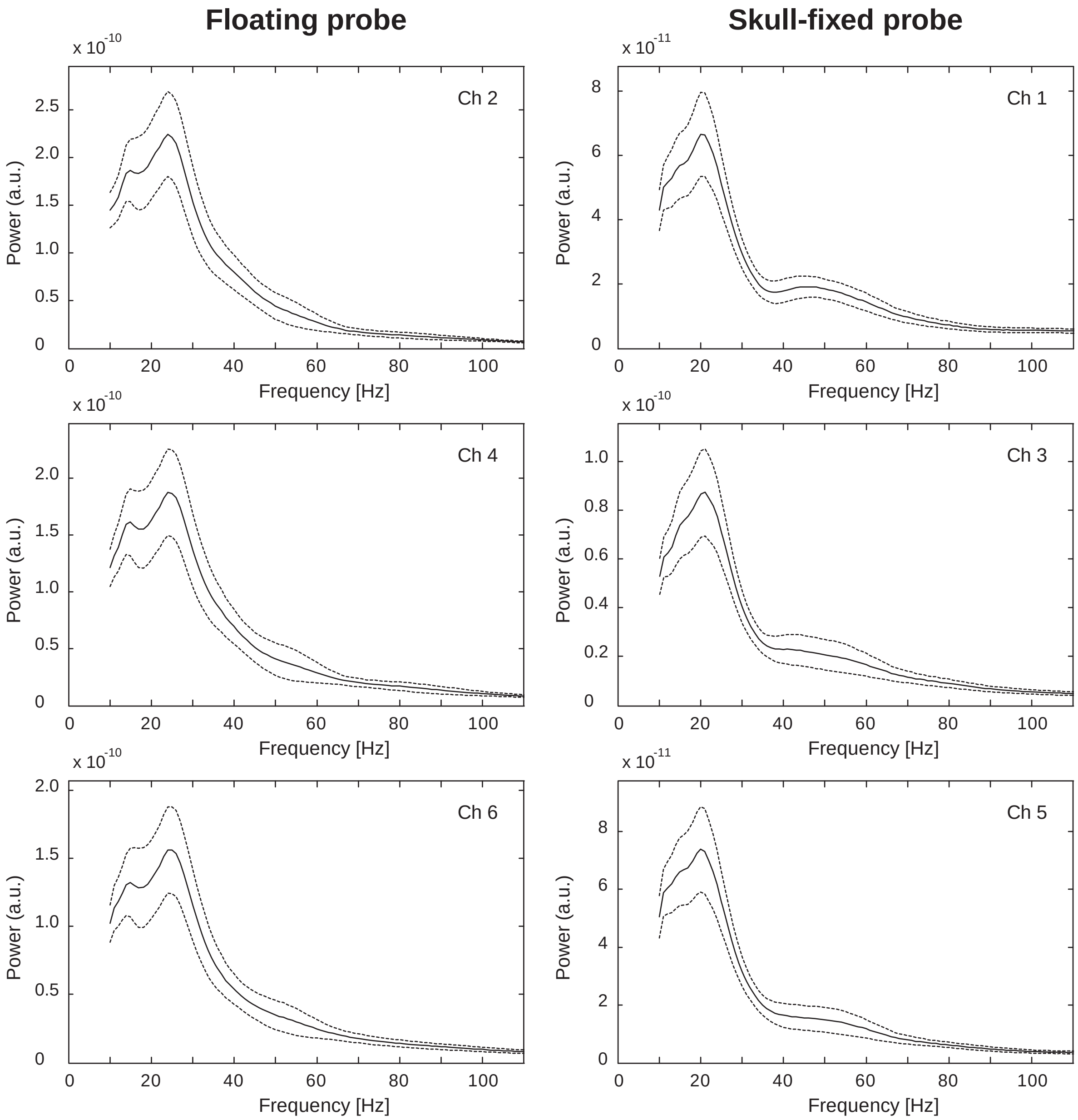

Supplemental Figure S1 | Powerspectra of the LFP signals recorded by means of the floating (left-side plots) and skull-fixed (right-side plots) silicon probes during the last week (i.e., Week 8) of recordings. These powerspectra were computed by averaging all trials (n = 53) of this recording session and quantify similarity measures between the signals recorded from randomly chosen channels. Power range (in a.u.) was computed for all channels and is displayed in Table S1. Note that the powerspectra from the signals recorded by means of both types of probes are very similar.
