# Supplementary material for: In vivo Recording Quality of Mechanically Decoupled Floating Versus Skull-Fixed Silicon-Based Neural Probes
Source: Front Neurosci. 2019 May 21;13:464. doi: 10.3389/fnins.2019.00464 (PMC6536660; doi:10.3389/fnins.2019.00464)
Supplement: Supplementary file 2 [file Table_1.pdf]

Table S1 | Amplitude range of LFP signals recorded by means of the skull-fixed vs. the floating probes.

	**Power range LFP signals** Week 8. All trials. (a.u.)	
	**Floating probe recorded signals** ($\times 10^{-12}$)	**Skull-fixed probe recorded signals** ($\times 10^{-12}$)
Minima	4.992	4.751
	6.512	4.278
	7.239	3.566
	4.792	2.621
	4.329	2.654
	4.581	2.814
	6.106	2.315
	8.687	2.241
	6.034	2.813
	7.054	2.661
	5.640	2.497
	4.193	2.991
	7.345	3.353
	4.360	3.672
	5.956	2.516
	6.385	4.002
**Average**	**5.888**	**3.109**
	**Floating probe recorded signals** ($\times 10^{-10}$)	**Skull-fixed probe recorded signals** ($\times 10^{-11}$)
Maxima	2.241	6.652
	1.876	8.731
	1.563	7.375
	1.970	8.960
	1.893	6.790
	2.131	6.149
	2.331	4.534
	1.436	7.814
	1.823	6.790
	1.574	6.922
	1.733	6.266
	1.363	6.636
	1.673	6.076
	1.349	7.323
	1.318	7.237
	1.890	6.264
**Average**	**1.760**	**6.907**
**Ratios max/min**	**29.891**	**22.216**

**Ratios min SFP / min FP**	**1.894**
**Ratios max SFP/ max FP**	**2.548**

SFP = Skull-fixed probe
FP = Floating probe
